# Use of Geospatial Modeling to Predict *Schistosoma mansoni* Prevalence in Nyanza Province, Kenya

**DOI:** 10.1371/journal.pone.0071635

**Published:** 2013-08-14

**Authors:** Dana M. Woodhall, Ryan E. Wiegand, Michael Wellman, Elizabeth Matey, Bernard Abudho, Diana M. S. Karanja, Pauline M. N. Mwinzi, Susan P. Montgomery, W. Evan Secor

**Affiliations:** 1 Division of Parasitic Diseases and Malaria, Centers for Disease Control and Prevention, Atlanta, Georgia, United States of America; 2 Geospatial Research, Analysis and Services Program, Centers for Disease Control and Prevention, Atlanta, Georgia, United States of America; 3 Centre for Global Health Research, Kenya Medical Research Institute, Kisumu, Kenya; Johns Hopkins University, United States of America

## Abstract

**Background:**

Schistosomiasis, a parasitic disease that affects over 200 million people, can lead to significant morbidity and mortality; distribution of single dose preventative chemotherapy significantly reduces disease burden. Implementation of control programs is dictated by disease prevalence rates, which are determined by costly and labor intensive screening of stool samples. Because ecological and human factors are known to contribute to the focal distribution of schistosomiasis, we sought to determine if specific environmental and geographic factors could be used to accurately predict *Schistosoma mansoni* prevalence in Nyanza Province, Kenya**.**

**Methodology/Principal Findings:**

A spatial mixed model was fit to assess associations with *S. mansoni* prevalence in schools. Data on *S. mansoni* prevalence and GPS location of the school were obtained from 457 primary schools. Environmental and geographic data layers were obtained from publicly available sources. Spatial models were constructed using ArcGIS 10 and R 2.13.0. Lower *S.mansoni* prevalence was associated with further distance (km) to Lake Victoria, higher day land surface temperature (LST), and higher monthly rainfall totals. Altitude, night LST, human influence index, normalized difference vegetation index, soil pH, soil texture, soil bulk density, soil water capacity, population, and land use variables were not significantly associated with *S. mansoni* prevalence.

**Conclusions:**

Our model suggests that there are specific environmental and geographic factors that influence *S. mansoni* prevalence rates in Nyanza Province, Kenya. Validation and use of schistosomiasis prevalence maps will allow control programs to plan and prioritize efficient control campaigns to decrease schistosomiasis burden.

## Introduction

Over 200 million people are infected with schistosomiasis, with approximately 85% of the burden of disease occurring in Africa [1], [2]. *Schistosoma mansoni*, one of the five schistosome species known to infect humans, can lead to significant morbidity, including anemia, malnutrition, and liver damage, and mortality. Preventative chemoprophylaxis, through mass distribution of praziquantel, is highly effective in decreasing disease burden. In endemic areas, neglected tropical disease (NTD) control programs rely on schistosomiasis prevalence rates to determine the target population and duration and frequency of preventative chemoprophylaxis distribution schedules [3]. Currently, the Kato-Katz technique, a procedure that requires microscopic examination of stool specimens and is both time and labor intensive, is the most widely used method to identify *S. mansoni* infection and the primary source of data for calculating infection prevalence [4], [5]. As the price of praziquantel has recently decreased and drug companies are increasing the quantity of drug donations, the bulk of costs incurred by schistosomiasis control program remain diagnosis and mapping of areas to determine prevalence of infection [6]. The development of a lower cost and more resource efficient method to accurately predict schistosomiasis prevalence would alleviate some of the financial constraints of NTD control programs.

In order for people to be at risk for *S. mansoni* infection, they must live in or visit an area where the environment is conducive for disease transmission. Disease transmission occurs in ecosystems that support both the snails that serve as the obligate intermediate host for the parasite and the parasite itself. Humans become infected through contaminated freshwater when free-swimming cercariae, which are released from the snail host, penetrate the skin; humans must have contact with contaminated freshwater in order to acquire *Schistosoma* infection [7]. Water bodies in which snail habitats flourish and people are exposed to the water correlate with areas of high human schistosomiasis prevalence [8].

The complex requirements for disease transmission contribute to the focal nature of schistosomiasis. Previous studies have examined environmental variables that affect snail habitation and parasite transmissibility such as soil pH, land surface temperature, and annual rainfall [9], [10]. Data regarding these geographical and climatic variables are continuously collected through satellite sensory systems and are freely accessible to the general public. Geographic, environmental, and climatic data have been used previously by researchers and NTD programs to create schistosomiasis prevalence maps based on known macroecological principles and parameters of snail and parasite survival. The development of such maps, utilizing geospatial modeling, has been used to predict disease prevalence rates for schistosomiasis throughout Africa [1].

Lake Victoria, which borders Nyanza Province, located in western Kenya, is the largest tropical lake in the world and is a known habitat of the freshwater snail genus *Biomphalaria* which serve as intermediate hosts for *S. mansoni* infection [11]. The lake is the main water source for people residing in Nyanza Province and is frequently used for bathing, washing, and fishing. As poor water and sanitation conditions exist throughout the area, people often defecate or urinate near or in the lake as they work or play. The ecologic features present in western Kenya create a permissive environment for both snail host reproduction and propagation of the schistosomiasis parasite to occur. The combination of a favorable snail habitat and frequent human contact with Lake Victoria places people who reside in the area at risk for *S. mansoni* infection. We sought to design a mixed model to assess the association between *S. mansoni* prevalence rates in schools and various environmental and geographical factors near Lake Victoria in western Kenya.

## Methods

### Ethics Statement

This study was reviewed and designated as non-research by the Institutional Review Boards at the Scientific and Ethical Review Committees of the Kenya Medical Research Institute (Kisumu, Kenya) and the Centers for Disease Control and Prevention. All data used in this study was data that had been previously collected for the Schistosomiasis Consortium for Operational Research and Evaluation (SCORE) project; written consent was obtained by participants and or their guardians on the behalf of children participants for their information to be stored in a database and used for research. All personal identifiers were removed prior to the receipt of the data used in this study. Institutional Review Board approval was granted for the use of the data prior to the start of the study.

### Study Site

This study was conducted in western Kenya in Nyanza Province, which borders the eastern edge of Lake Victoria. Schools from the following seven districts were included in the study: Bondo, Kisumu, Nyando, Rachuonyo, Homa Bay, Suba, and Siaya. No mass drug administration of chemoprophylaxis treatment for schistosomiasis had been initiated in the Province prior to the start of the study.

### Disease Prevalence Data

*S. mansoni* prevalence data were collected through the SCORE project, a large multi-country study dedicated to research to guide schistosomiasis control and elimination programs [12]. School-level prevalence data were collected from 457 primary schools located in Nyanza Province from September through December 2010. School children, ages 10–18 years, were included in the study. *S. mansoni* prevalence was calculated by examining two slides from a single stool specimen from each participant using the Kato-Katz method to identify schistosome eggs [13]. Each stool sampled was qualitatively read for schistosome eggs and recorded as positive or negative. If a child had at least one schistosome egg on either slide, he or she was counted as positive. To calculate the schistosomiasis prevalence for each school, the number of positive children was divided by the total number of children tested.

### School Geospatial Data

For each primary school, school name and geographic coordinates, including latitude, longitude, and altitude, were electronically captured during site visits using handheld global positioning system (GPS) units (Trimble Navigation Ltd, California, USA). For schools for which GPS coordinates were not collected or incompletely recorded, geographic coordinates were obtained by manually searching for the school name using the United States Geological Survey’s Earth Explorer (http://edcsns17.cr.usgs.gov/EarthExplorer/).

### Geographic and Environmental Data

Geographic, population, climatic, and environmental data were obtained from various publicly accessible remote sensing data sources. Basic topographical land maps of Nyanza Province were accessed through the Kenya Medical Research Institute located in Kisumu. Data for soil parameters, including soil bulk density (g/cm^3^), total soil water capacity (cm/m), pH, and texture class (fine, medium, or course) were obtained from the International Soil Reference and Information Centre (ISRIC) World Soil Information website and were derived from the Soil Terrain Database for Kenya (KENSOTER) at a scale of 1∶1M [14]. Average monthly rainfall data (mm) was obtained from the U.S. Agency for International Development (USAID) Famine Early Warning Systems Network (FEWS NET) at 8 km resolution [15]. Human influence index (HII), was extracted from the Socioeconomic and Data and Applications Center (SEDAC) database at 1 km resolution [16]. HII calculates changes in the environment due to anthropometric activities. The 8 variables used to calculate HII include population density, railroads, major roads, navigable rivers, coastlines, nighttime stable lights values, urban polygons, and land cover categories. HII is based on a scale from 0 to 64, with 64 representing the maximum human influence. Normalized difference vegetation index (NDVI) (a proxy for vegetation), land use, and day and night land surface temperature (LST) (°C) data came from the Land Processes Distributed Active Archive Center [17]. Land use categories included agricultural, bush land, plantation, swamp and town. Population distribution data for 2010 were downloaded from the Oak Ridge National Laboratory Geographic Information Science and Technology website [18]. Distance to Lake Victoria (km) was measured as a straight line between the school and the shoreline using ArcGIS version 10.0 (Environmental Systems Research Institute, California, USA 2011).

Spatial analysis was conducted in ArcGIS version 10.0. Vector data attributes were linked to primary schools via spatial joins, and for raster data, cell values were attributed to primary schools by extraction. When distance calculations were necessary, vector layers were projected to UTM (Universal Transverse Mercator) zone 36S, which corresponds to the location of Kenya; distances were computed in meters. All other data were analyzed in the WGS84 (World Geodetic System) coordinate system.

Rainfall, LST, and NDVI were aggregated over the study period. When a school location possessed a missing value in the rainfall layer, it was assumed zero rain had accumulated during that period. Rainfall was then summed over the twelve decadal (120 days) comprising the study period and converted to a monthly average. LST values of zero or less were treated as missing values. All available values were then averaged and converted from Kelvin to Celsius. For one school, all NDVI values were less than zero and were recorded to the nearest pixel with all values greater than zero. Non-missing NDVI values for each school were averaged. All conversions and scaling modifications were completed in accordance with the Land Processes Distributed Active Archive Center data download guidelines [17]. As the majority of land in Nyanza Province is agricultural, the land use variable was condensed into 2 variables, agricultural and non-agricultural land use (which included bush land, plantation, swamp, and town categories) for data analysis.

All study predictors with continuous or count distributions, excluding HII, were categorized into quartiles to avoid overly restrictive linear assumptions. The majority of school locations in this study possessed HII values of 18 or 22; logical groupings for the HII were 18, 22 and greater than 26.

### Statistical Analysis

All statistical tests were two-sided and use a 5% level of significance. R version 2.13.0 (R Core Development Team 2011) was used for all analyses [19].

Semivariograms were created to assess the level of spatial clustering in *S. mansoni* rates. After relevant covariates were included, semivariances were consistent across distance and direction suggesting that an isotropic model was sufficient.

Univariable, quasi-Poisson regression models were run to explore associations between *S. mansoni* rates and all potential predictors and account for any overdispersion in the data [20]. The outcome in each model is the number of *S. mansoni* infected children with the number of children sampled included as an offset. In non-spatial models, an automated variable selection procedure using the Bayesian Information Criterion was employed to arrive at a final, multivariable model [21], [22]. This same model was also found to have the lowest quasi-AIC (qAIC) from the MuMIn package [23]. Variables that did not improve model fit were excluded from the final multivariable model. We found an improvement in qAIC of 870.95 for the multivariable model compared to the model with only an intercept and the offset. Using the same variables as the multivariable model, a generalized linear mixed model employing an exponential correlation function was fit using the glmmPQL function with location specific random effects to control for spatial correlation in the multivariable model [24]. After using a quasi-likelihood approach to account for overdispersion the spatial component had a negligible effect on the standard errors. Hence, the non-spatial multivariable and spatial multivariable models were largely similar.

## Results

**Figure 1:**
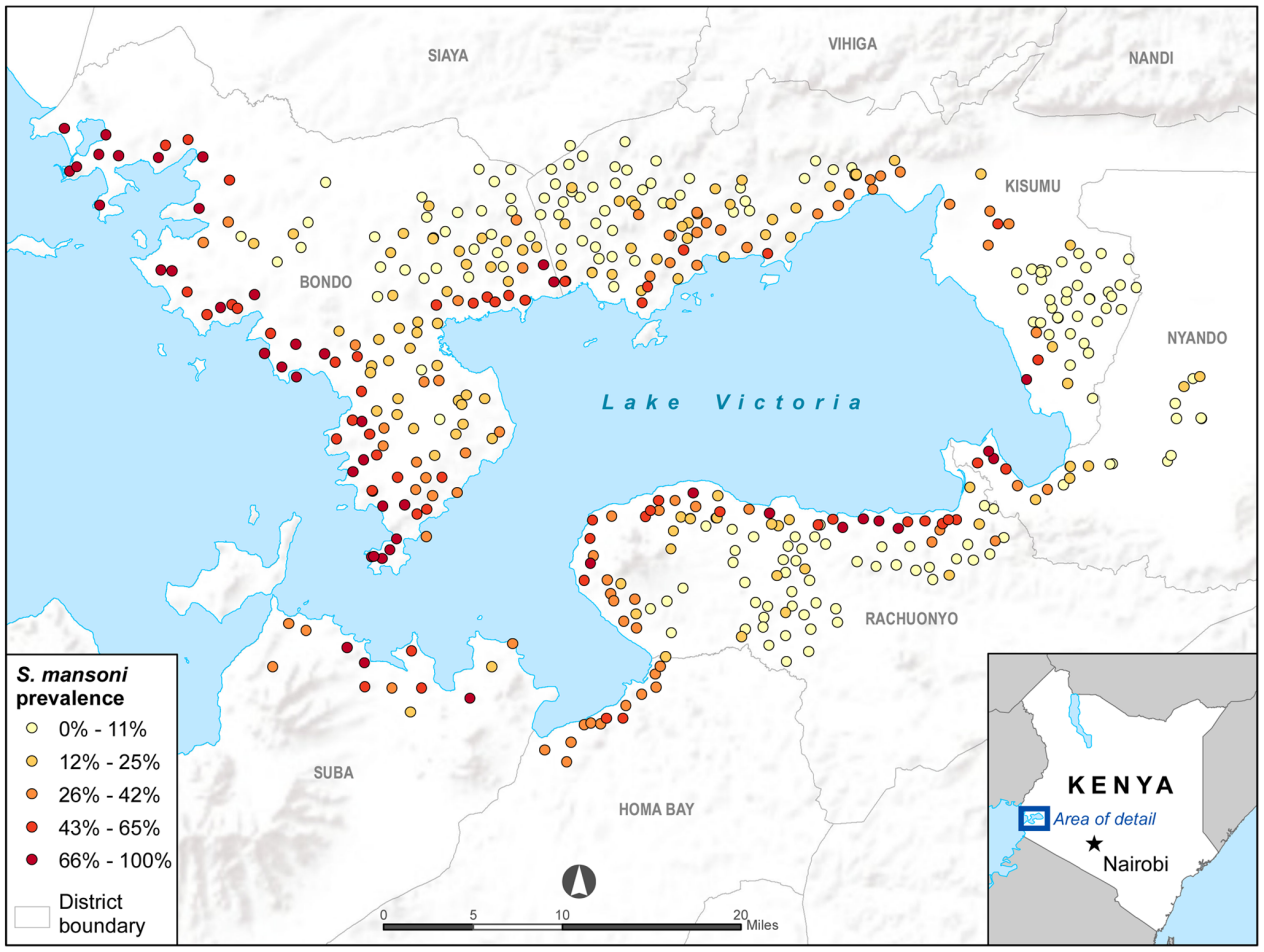
*S. mansoni* prevalence in selected primary schools in Nyanza Province, Kenya.

**Figure 2:**
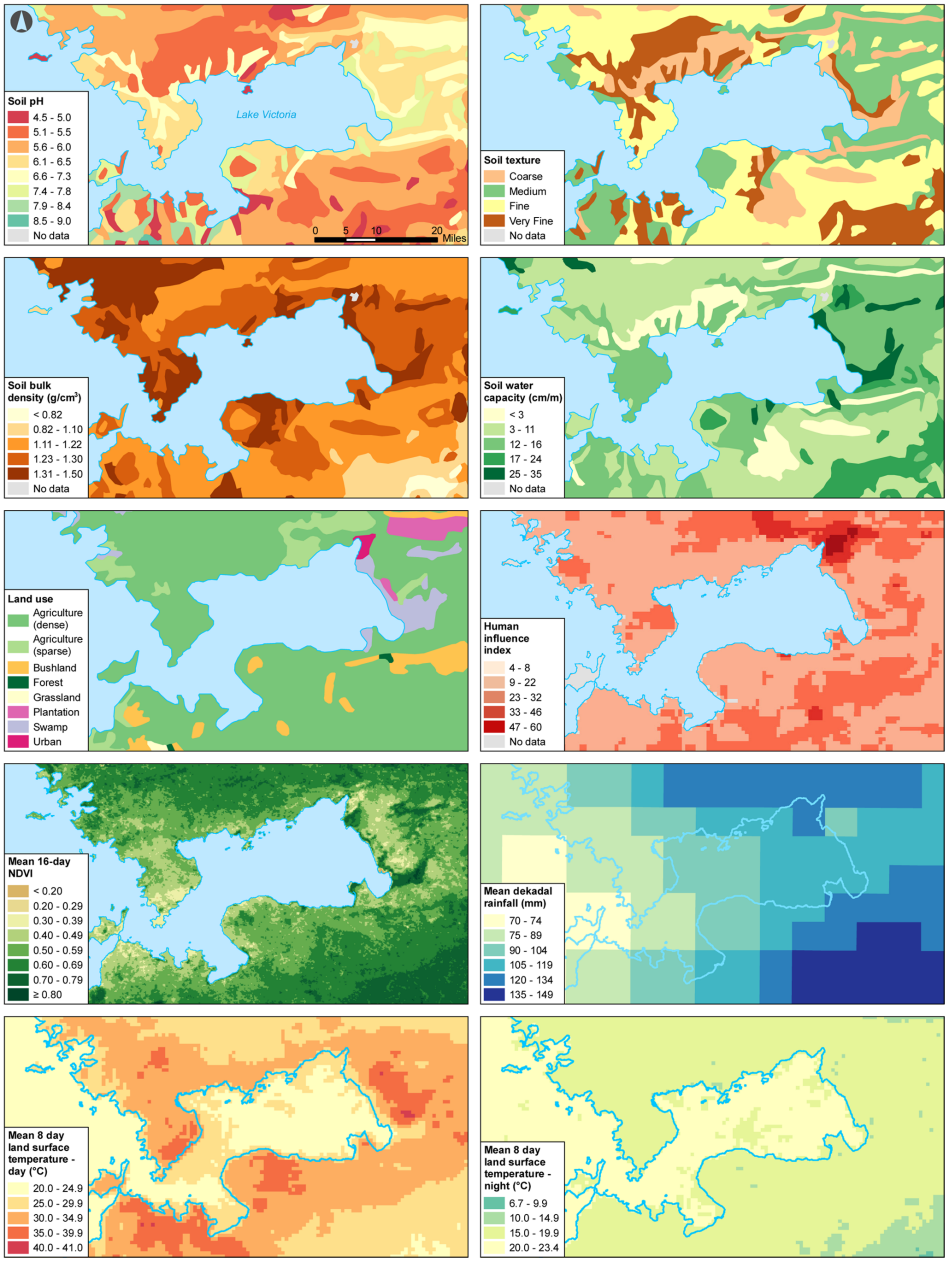
Spatial distribution of environmental and geographic variables for Nyanza Province, Kenya.

The mean *S. mansoni* prevalence for the 457 primary schools in Nyanza Province included in our study was 17% (range 0–100%).The mean number of children tested per school was 42 (range 15–71 students). Children infected with *S. mansoni* were found in 426 of the 457 visited schools. Figure 1 shows *S. mansoni* prevalence rates and location for each school; schools closest to the border of Lake Victoria had the highest prevalence rates. Maps (Figure 2) were constructed for the following 10 variables: soil pH, soil texture, soil bulk density, total soil water capacity, land use, HII, NDVI, monthly rainfall, day LST, and night LST.

**Figure 3:**
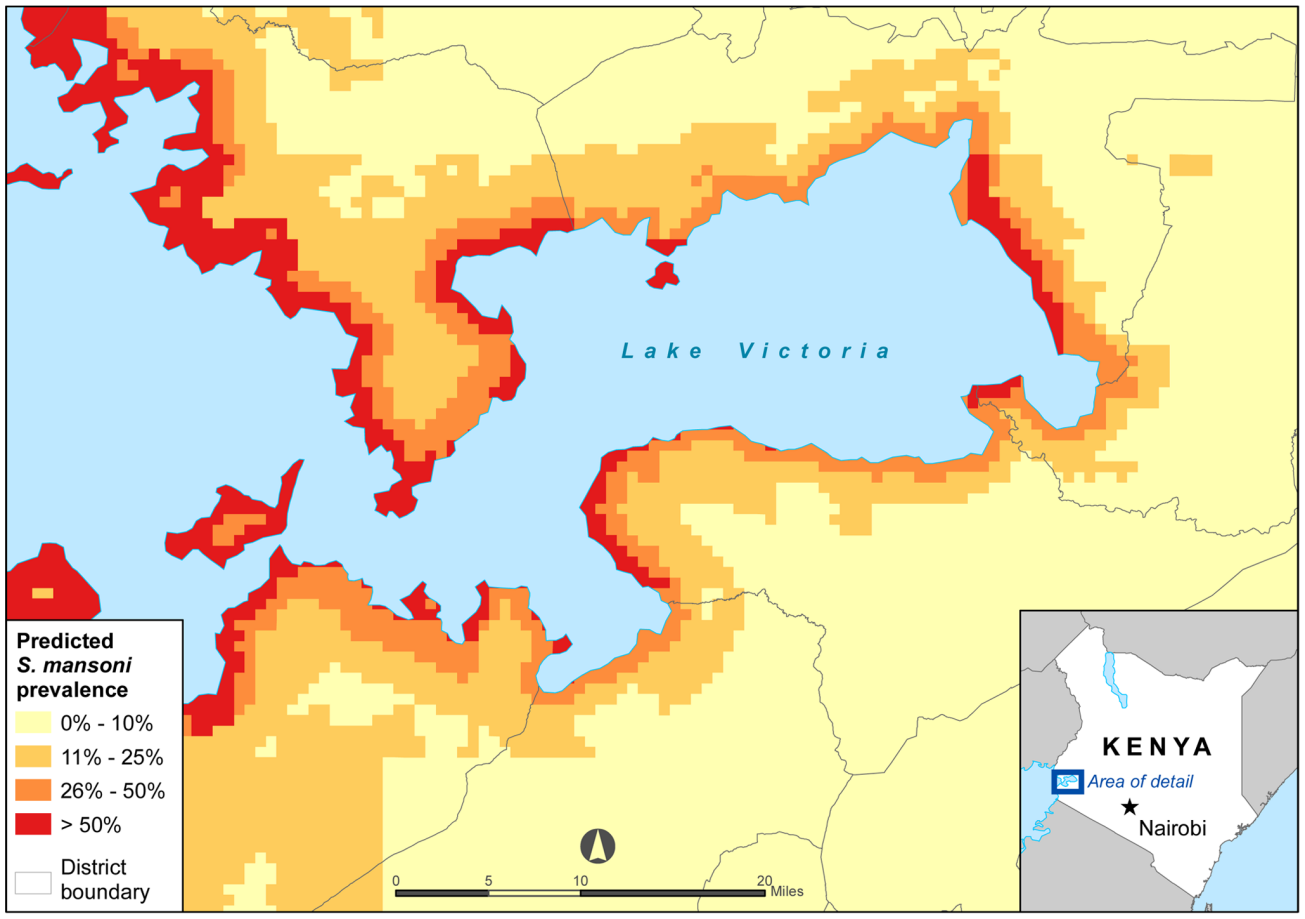
Predicted *S. mansoni* prevalence in Nyanza Province, Kenya.

Table 1 gives the prevalence ratios (PR) and 95% confidence intervals (CI) using univariable and multivariate non-spatial logistic regression and multivariate spatial logistic regression. Spatial modeling results show a significant decrease in *S. mansoni* prevalence with increased distance of the school from Lake Victoria. Day LST and monthly rainfall were also significantly associated with *S. mansoni* prevalence. Increasing day LST from 30.382°C to over 34.310°C was correlated with a decrease in *S. mansoni* prevalence. Higher monthly rainfall totals were associated with lower *S. mansoni* prevalence rates. All three variables, distance to Lake Victoria, day LST, and monthly rainfall had significant associations with *S. mansoni* in both in the univariable and multivariable models. The remaining analyzed variables, including altitude, soil pH, soil texture, soil bulk density, total soil water capacity, population, land use, HII, NDVI, and night LST, did not improve the multivariate model’s fit to the data and therefore were excluded from the final model. Based on the data from the final model, a map was created to show the predicted *S. mansoni* prevalence in Nyanza province (Figure 3).

**Table 1:** Results of univariable and multivariable Poisson regression analyses for *S.mansoni* summarized by prevalence ratios (PR) and 95% confidence intervals (CI).

**Night LST (°C)**≤17.41017.423–18.11018.115–18.524>18.524	0.77(0.60,0.98) 0.0382 0.77(0.60,0.98) 0.0355 1.13(0.91,1.41) 0.2803		
**NDVI** 2≤0.465700.46571–0.512830.51328–0.56498>0.56498	0.90(0.73,1.11) 0.3190 0.65(0.51,0.81) 0.0002 0.52(0.41,0.67) <0.0001		
**Human Influence Index**1822>26	0.75(0.62,0.91) 0.0034 0.69(0.54,0.87) 0.0023		
**Rainfall (monthly, mm)**≤1.311.33 - 2.993.00 - 6.10>6.10	0.80(0.70,0.91) 0.00080.56(0.49,0.64) <0.00010.47(0.40,0.56) <0.0001	0.80(0.70,0.91) 0.00080.56(0.49,0.64) <0.00010.47(0.40,0.56) <0.0001	0.80(0.70,0.91) 0.00080.56(0.49,0.64) <0.00010.47(0.40,0.56) <0.0001
**Soil pH**≤5.35.4–6.36.4–6.5>6.5	2.09(1.65,2.66) <0.00011.60(1.22,2.09) 0.00061.80(1.39,2.34) <0.0001		
**Soil texture class**CoarseFineMedium FineVery Fine	1.68(1.33,2.13) <0.0001 1.19(0.93,1.53) 0.1738 0.99(0.75,1.31) 0.9698		
**Soil bulk density (kg/dm^3^)**≤1.211.22–1.301.33>1.33	1.09(0.88,1.37) 0.4260 1.38(0.87,2.10) 0.1502 1.91(1.52,2.41) <0.0110		
**Soil water capacity (cm/m)**≤89–1213–14>14	0.51(0.40,0.64) <0.0001 1.06(0.87,1.28) 0.5563 0.68(0.51,0.91) 0.0110		
**Population, 2010 (people per cell(1** **km×1** **km))**≤7374–226228–424>424	0.68(o.54,0.85) 0.0011 0.62(0.49,0.79) <0.0001 0.75(0.60,0.93) 0.0099		
**Agricultural land use**NoYes	0.74(0.50,1.17) 0.1686		
**Altitude (m)**≤11531153.3–1180.01180.1–1212.11>1212.1	1.10(0.88,1.36) 0.4117 0.95(0.76,1.19) 0.6523 0.56(0.43,0.72) <0.0001		

1 Land surface temperature.

2 Normalized difference vegetation index.

## Discussion

Determining *S. mansoni* prevalence in areas where disease transmission occurs is the core starting point for schistosomiasis control programs; all subsequent planning and implementation strategies are based off these initial prevalence values. Utilizing environmental, climatic, and geographic data to identify locations that have the highest likelihood of schistosomiasis transmission can help schistosomiasis programs focus their control efforts on populations living in high risk areas. Previous studies that have generated *S. mansoni* spatial risk maps on both the continental and country level have acknowledged limitations in attempting to use these maps to initiate disease control measures in specific geographic locations [25]. One concern is that these large maps do not accurately capture environmental differences between various ecologic zones making prevalence prediction less precise. Areas such as Lake Victoria, which have unique and diverse ecologic components, are especially difficult to fit into large scale geostatistical models; researchers who recently constructed a statistical model to predict *S. hematobium* prevalence for all of Tanzania found that their model performed poorly in areas near Lake Victoria and attributed this difference to the unique environment around the lakeshore [26]. To our knowledge, this is the first study to use geospatial models to identify environmental variables which specifically affect the *S. mansoni* prevalence rate in Nyanza Province, Kenya. Although similar modeling has been completed previously in Kenya, our work was able to identify prevalence differences on a smaller scale which were previously undetected by geospatial modeling that was performed on a larger scale [27]. This may have important implications for control programs as *S. mansoni* prevalences in specific areas may be incorrectly estimated by large scale mapping models leading to areas potentially being over or undertreated with mass drug distribution.

Our spatial model found that three variables, distance to Lake Victoria, average rainfall, and day LST were significantly associated with *S. mansoni* prevalence. The strongest predictor of higher *S. mansoni* prevalence rates was distance to Lake Victoria; the closer a primary school was to Lake Victoria, the higher the *S. mansoni* prevalence rate in the students tested for the infection. This finding is not surprising as children who live closer to the lake are more likely to have contact with water on a regular basis [28]. Research has demonstrated that using a 5 km ceiling from the lakeshore to local areas schools would accurately classify *S. mansoni* prevalence in 90% of the schools and that schools with >50% prevalence were located within 1.5 km of the lakeshore. A separate study showed that schools at distances greater than 5 km had *S. mansoni* prevalence was consistently <15% [29]. The significant association between school distance to Lake Victoria and *S. mansoni* prevalence rates highlights the importance accessibility to potential *S. mansoni* infested water sources in determining the likelihood of infection in at risk populations.

In addition to human proximity to fresh water sources, our study found that environmental factors also directly influence *S. mansoni* prevalence rates in humans. Climatic variables, specifically rainfall and temperature, are known to be associated with differences in snail population size and infection rates [30]. A previous study around Lake Victoria in Uganda established that the *S. mansoni* prevalence rate was absent in areas receiving <900 mm of rainfall annually [31]. As the amount of annual rainfall amount throughout Nyanza Province ranges from 900 mm to upwards of 1600 mm, our study area can be defined as a hospitable environment for *S. mansoni* transmission [32]. Although rainfall is linked to increasing *S. mansoni* prevalence rates, our study findings indicate that excessive amounts of rainfall are associated with lower *S. mansoni* rates. While rainfall promotes the continuous presence of standing water bodies and contributes to the growth of vegetation which enable sustainable snail development, too much rainfall may create a less hospitable environment for snail development based on their established macroecological characteristics. *Biomphalaria* spp thrive in shallow water (0–7 cm deep) along the shoreline in areas of low water velocity (optimum 13.3 cm/s, range 12–21) [33]. Large amounts of rainfall may alter the environment enough to disrupt snail habitats and therefore affect *S. mansoni* transmission. Changes in human behavior due to excessive rainfall may offer another possible explanation for our findings. In areas of extremely high rainfall, new standing water pools may be established and people may use these fresh water areas to carry out bathing or washing activities, decreasing contact time with Lake Victoria; these newly created water sources are less likely to be inhabited by snails.

We also found a significant inverse relationship between *S. mansoni* prevalence rates and increasing day LST; the higher the temperature the lower the *S. mansoni* prevalence. The average day LST in our study area fell within a fairly narrow temperature range with the majority of temperature values falling between approximately 30°C and 34°C. Numerous macroecological studies have been carried out in an effort to determine the optimal temperature range for both snail survival and transmission of the schistosome parasite. Researchers in the 1980s published studies revealing that *B. pfeifferi* snails infected with *S. mansoni* do not survive outside of a temperature range from 16 to 30°C [33], [34]. Another study that examined the number of eggs hatched from *B. pfeifferi* living at various temperatures found that the maximum temperature for egg production occurred between 30 and 35°C with no eggs hatching above 35°C [35]. It should be noted that these studies occurred in the laboratory; in the natural environment temperature variations exist and snails are able to migrate to areas where more optimal temperatures occur. Applying this knowledge to our findings, the significant decrease in *S. mansoni* prevalence with respect to increasing temperature is a logical finding as temperatures around 34°C are at or near the upper limit for snail survival.

Our findings that day LST and rainfall significantly affects *S. mansoni* prevalence highlight the need for further studies exploring the relationship between climatic variables and *S. mansoni* transmission. The establishment of seasonal patterns that affect *S. mansoni* transmission could have direct implications for determining the optimal schedule for chemoprophylaxis distribution. Defining seasonal and climatic patterns could also influence the timing of efforts aimed at controlling snail populations through the use of chemical agents. It is important to note that our study links specific environmental variables with schistosomiasis prevalence during a defined time period and does not necessarily account for the environmental conditions present at the time when infection was acquired.

As access to the internet is becoming more readily available and mapping software is improved, the use of geospatial modeling to predict schistosomiasis prevalence rates is increasingly recognized as important tool in understanding disease transmission and identifying at risk populations. There are numerous benefits to using geospatial modeling to determine *S. mansoni* prevalence rates. First, geographic areas where *S. mansoni* transmission may occur can be defined based on known biologic and environmental parameters necessary for both the parasite and the associated snail vector. Over time, environmental and climate changes can be reassessed and used either to determine new areas where *S. mansoni* may occur or delineate areas where *S. mansoni* transmission may no longer occur due to the emergence of inhospitable environments. Maps can also be quickly reconstructed, without great additional financial resources, to accurately capture quick population movements, such as the establishment of a refugee camps, or new construction projects, which may alter access to water resources such as dams. As schistosomiasis control programs are working within the constraints of limited funding to carry out activities, geospatial modeling could provide them with a cost efficient method to determine areas where they should focus their control efforts.

Our study has a number of limitations. First, the data used in our spatial model is aggregated data and therefore we are unable to account for individual behaviors which may directly affect schistosomiasis transmission and prevalence rates. Another limitation is the fact that our study occurred in a relatively homogenous area, which meant that there was little variability within several of the environmental, geographic, and socioeconomic variables, including altitude and HII, incorporated into our model. While some of these variables may affect schistosomiasis transmission, our model may not have detected the effects due to lack of variability within our study site. Lastly, our *S. mansoni* prevalence data rely on the accuracy of Kato-Katz tests performed on stool samples from the enrolled school children. Although this test is a widely accepted method for determining *S. mansoni* prevalence rates, it is relatively insensitive. Incorrect estimates of school schistosomiasis prevalence rates would lead to inaccuracies in our model.

Further studies would help us to gain a clearer understanding of the interaction between environmental, human, snail vector, and parasite features in Nyanza Province. Research aimed at investigating year to year schistosomiasis transmission would enable us to better define which environmental factors are strongly correlated with higher *S. mansoni* prevalence rates over time. Because no previous mass treatment for schistosomiasis was administered in our study area, our model represents the lifetime aggregation of infection in the study participants and may thereby underrepresent some of the relevant ecologic factors in the final statistical model. Complete mapping of snail habitats around the Kenyan border of Lake Victoria would assist us in defining conditions that permit snail habitats to thrive and are conducive for parasite transmission to occur. Incorporating *S. hematobium* and soil-transmitted helminthes prevalence data into the model would help to gain an understanding of how the prevalence of other parasitic diseases is influenced by environmental and geographic variables. Validation of our model in other settings is needed to determine if our results our generalizable in other schistosomiasis endemic areas.

Our research has demonstrated that specific environmental and geographic variables, distance to Lake Victoria, day LST, and monthly rainfall, are significantly associated with *S. mansoni* prevalence in Nyanza Province, Kenya. Maps generated from models that incorporate geographic and environmental data can provide schistosomiasis control programs with a cost effective tool to determine areas where they should focus control program efforts. Implementation of efficient control campaigns will ultimately lead to a decrease in human morbidity and mortality due to schistosomiasis.
